# A Qualitative Study of Knowledge of Metabolic Syndrome, Attitudes about Lifestyle Modifications, and Preferences for Lifestyle Interventions among Patients with Cancer and Metabolic Syndrome

**DOI:** 10.21203/rs.3.rs-3232672/v1

**Published:** 2023-09-04

**Authors:** Isabel Martinez Leal, Ashwathy B. Pillai, Jessica T. Foreman, Kimberly W. Siu, Natalia I. Heredia, Carmen Escalante, Ellen F. Manzullo, Aimee J. Christie, Tamara E. Lacourt, Zayd A. Razouki, Jessica P. Hwang

**Affiliations:** The University of Texas MD Anderson Cancer Center; The University of Texas MD Anderson Cancer Center; The University of Texas MD Anderson Cancer Center; University of Washington; The University of Texas Health Science Center at Houston; The University of Texas MD Anderson Cancer Center; The University of Texas MD Anderson Cancer Center; The University of Texas MD Anderson Cancer Center; The University of Texas MD Anderson Cancer Center; The University of Texas MD Anderson Cancer Center; The University of Texas MD Anderson Cancer Center

**Keywords:** metabolic syndrome, cancer, healthy lifestyle, qualitative research

## Abstract

**Background:**

Nearly 60% of patients with cancer have metabolic syndrome, which increases the risk of mortality, but there is no clear guidance for oncology providers about its management. Here, we report on the qualitative component of a larger mixed methods study that aimed to understand cancer patients’ knowledge, attitudes, and preferences regarding metabolic syndrome.

**Methods:**

Adult cancer patients with metabolic syndrome were recruited during 2022–2023 in the MD Anderson General Internal Medicine clinic and participated in semistructured interviews focused on metabolic syndrome and lifestyle interventions. Interviews were audio-recorded and transcribed verbatim. Participants’ demographic information was collected. Interviews were analyzed using hybrid thematic analysis and constant comparison involving deductive and inductive coding. Researcher triangulation and debriefing were used to ensure rigor.

**Results:**

There were 19 participants, 12 female and 12 White. Eighteen had solid tumors, including gynecologic (n = 5), genitourinary (n = 4), colorectal (n = 3), and breast (n = 2). Analysis yielded 5 major themes: 1) patients’ understanding of metabolic syndrome; 2) attitudes about and approaches to managing metabolic syndrome; 3) capacity and limitations regarding managing metabolic syndrome; 4) patient-led care; and 5) tailored intervention plans. Participants had limited knowledge of metabolic syndrome and its cancer-related consequences; most desired additional education. Many participants reported that their cancer or diabetes diagnosis motivated them to prioritize lifestyle Modifications. Participants expressed strong interest in personalized care plans focused on healthy lifestyle rather than simply weight loss. As part of their tailored intervention plans, participants desired clear communication with their medical team, coordination of care among team members, and collaboration with providers about treatment decisions.

**Conclusion:**

Cancer patients with metabolic syndrome want collaborative, patient-centered care. Shared decision-making based on respect for patients’ distinctive needs and preferences is an essential component of the development of such collaborative care. Tailored interventions, practical implementation strategies, and personalized care plans are needed for cancer patients with metabolic syndrome. The study findings contribute to filling the gap in knowledge regarding clear guidance for oncology providers on managing metabolic syndrome and will inform the development of future lifestyle interventions for patients diagnosed with metabolic syndrome.

## INTRODUCTION

Metabolic syndrome is characterized by hypertension, dyslipidemia, insulin resistance, and obesity and is defined by the presence of at least 3 of the following 5 components: elevated waist circumference, elevated triglyceride levels, reduced high-density lipoprotein cholesterol level, elevated blood pressure, and elevated fasting glucose level.^1^ Metabolic syndrome affects nearly 35% of US adults, and the prevalence increases with age, such that metabolic syndrome affects nearly 50% of individuals 60 years and older,^2^ a group at heightened risk for cancer.^3^ Metabolic syndrome increases the risk of developing multiple cancers, including colorectal cancer (odds ratio [OR] 1.22, 95% CI 1.20–1.24),^4^ breast cancer (hazard ratio [HR] 1.13, 95% CI 1.00–1.27),^5^ endometrial cancer (HR 1.39, 95% CI 1.32–1.47),^6^ hepatocellular cancer (OR 2.13, 95% CI 1.96–2.31),^7^ and cholangiocarcinoma (OR 1.56, 95% CI 1.32–1.83).^7^

Among patients with cancer, the prevalence of metabolic syndrome has been estimated to range from 24% among patients with urothelial cancer^8^ to 34% among those with breast cancer^9^ to 60% among those with endometrial cancer.^10^ Metabolic syndrome has been associated with an increased risk of cancer-specific mortality among patients with colon cancer (relative risk 1.72, 95% CI 1.03–2.42),^11^ gastrointestinal cancer (esophageal, gastric, or colorectal) (HR 1.64, 95% CI 1.18–2.28),^12^ breast cancer (HR 1.73, 95% CI 1.09–2.75),^13^ endometrial cancer (HR 1.28, 95% CI 1.09–1.53),^14^ and urothelial cancer (HR 1.38, 95% CI 1.01–1.89).^8^ Metabolic syndrome has also been associated with increased risk of recurrence among patients with colon cancer (HR 2.11, 95% CI 1.23–2.81)^15^ and with progression from castration-sensitive to castration-resistant prostate cancer (HR 1.41, 95% CI 1.09–1.81).^16^

Despite the growing body of evidence showing the harmful impact of metabolic syndrome on cancer development and clinical outcomes, there is no clear guidance for oncology providers regarding the management of metabolic syndrome. National oncology recommendations have focused on obesity and weight loss.^17–19^ Oncology providers often do not ask patients about their diet,^20^ so it is unclear what patients know about metabolic conditions and what patients with metabolic syndrome need from their medical team to learn how to manage this syndrome. In this study, we interviewed patients with cancer and metabolic syndrome to understand their knowledge of metabolic syndrome, attitudes about lifestyle modifications, and preferences for lifestyle interventions. The results can ultimately be used to inform the development of future patient-centered care interventions.

## METHODS

### Study Design, Participants, and Recruitment

The current study reports the findings of the qualitative component of a larger mixed methods project. As little is known about cancer patients’ experiences and perspectives of metabolic syndrome, an exploratory qualitative approach was adopted. Our focus was on understanding and responding to the lifestyle modification needs and preferences of cancer patients diagnosed with metabolic syndrome. Findings from the mixed methods study will be reported separately and will be used to inform the development of future lifestyle interventions to guide physicians and patients with cancer on managing metabolic syndrome. This study was reviewed and approved by the Institutional Review Board of The University of Texas MD Anderson Cancer Center prior to study commencement.

The target sample for this study was 20 participants. In qualitative research, the specific research questions guide the study design, sample size and sampling strategy, data collection methods, and data analysis methods.^21,22^ (Green & Thorogood, 2010; Renjith et al, 2021) As this was a well-defined and structured study that addressed specific research questions using an interview format in which participants were all asked the same questions, we anticipated that saturation would be reached at 20 participants or fewer.^23^ Saturation is the point at which data analysis no longer yields any new information or themes, producing only redundant findings.^24^

Working closely with providers and clinic staff, the research team used purposeful sampling^25^ to recruit participants between June 2022 and February 2023 from the MD Anderson General Internal Medicine Clinic. To be eligible, individuals had to be ≥ 18 years old, have a history of cancer, have been diagnosed with metabolic syndrome by a General Internal Medicine provider, and speak English. Potential participants were recruited through various means, including emails sent through the electronic medical record, phone calls, and provider and staff notification about the study. Individuals who expressed interest in participating were screened for eligibility by members of the research team. To maintain privacy, potential participants were contacted using secure messaging through the electronic medical record and using the Skype teleconferencing platform. Participants provided written consent through the electronic medical record prior to their engagement in the research. Each participant received a $25 gift card after completion of the interview and other protocol requirements, including a brief demographic survey.

### Data Collection

A semistructured interview guide (see Appendix) was developed based on a literature review as well as the research team’s expertise with treating metabolic syndrome among cancer patients and conducting qualitative research. Three team members in the Department of General Internal Medicine—a clinical research fellow (ABP), a program manager (JTF), and a research coordinator (KWS)—conducted individual interviews using the semistructured interview guide, with participants using the Zoom videoconferencing platform. Interviews generally lasted approximately 60 minutes. Prior to starting the interviews, interviewers discussed the nature of the study and the scope of the interview questions with participants. Interviews were audio-recorded and were later transcribed verbatim by a professional transcription service. To safeguard participants’ privacy, transcripts were deidentified and stored in a password-protected database along with audio-recordings. Questions focused specifically on discerning patients’ understanding of and attitudes about metabolic syndrome and how this condition can affect cancer outcomes, what patients needed from their medical team to learn how to manage their metabolic syndrome, patient-identified facilitators of and barriers to adopting behavioral change, and preferences for future interventions. Demographic information was collected through a survey and from the electronic medical record.

### Data Analysis

A hybrid approach to thematic analysis was used involving deductive and inductive coding. This is a flexible analytic approach that entails an ongoing and iterative process of analysis based on the constant comparison method.^26^ Initially, deductive coding was used. Predetermined codes derived from the research aims, relevant literature, and the interview questions were used to develop a codebook consisting of codes with their definitions and criteria for use.^27^ Transcripts were coded using the predetermined codes by a team of multidisciplinary researchers with expertise in general internal medicine (JPH, ABP) and cultural anthropology and public health (IML). To limit researcher bias, all transcripts were divided between the 3 main analysts (JPH, ABP, IML), and each transcript was independently coded by at least 2 analysts. The research team met weekly to review codes, note any emerging codes, and discuss coding until agreement was reached on the codebook. After team coding and review of the initial 10 transcripts, analysts began inductive coding to refine existing codes and develop new codes and categories drawn from the data. KWS assisted with final coding, analysis, and importing the dataset into Dedoose (version 9.0.90, Los Angeles, CA: Sociocultural Research Consultants, LLC) to organize the data. Constant comparison was used to refine codes, confirm definitions, avoid redundancy, ensure accurate accounting of all the data, and substantiate attainment of saturation.^28^ Analytic rigor was ensured through a process including researcher triangulation, reviewing and refining of codes through team coding, accurate accounting of all the data, and a final team discussion to ensure the analysis fit the data. The various analytic techniques used by the research team to ensure rigor of findings were in accordance with Guba’s seminal work^29^ on the evaluative criteria for assessing the trustworthiness of qualitative research, through establishing transferability, or external validity; dependability, or reliability; credibility, or internal validity; and confirmability, or objectivity.

## RESULTS

Nineteen individuals participated in the study, most of them female and non-Hispanic White (Table 1). Although the target sample size for this study was 20 participants, thematic saturation was reached with these 19 participants, who expressed consistency across their responses.^30^ Attainment of thematic saturation was confirmed through the failure of additional data analysis to generate any new information or themes, rendering continued interviewing redundant. Thematic analysis yielded 5 major themes: (1) patients’ understanding of metabolic syndrome; (2) patients’ attitudes about and approaches to managing metabolic syndrome; (3) patients’ capacity and limitations with respect to managing metabolic syndrome; (4) patient-led care; and (5) tailored intervention plans. The themes focus on patients’ experiences and care preferences regarding metabolic syndrome, as well as provider recommendations. Table 2 shows themes and associated categories along with example quotes. Pseudonyms are used for the sources of the direct quotations to protect patient privacy.

### Theme 1: Patients’ Understanding of Metabolic Syndrome

When asked about their understanding of metabolic syndrome, patients gave responses indicating (1) *unfamiliarity with metabolic syndrome*; (2) *desire for education about metabolic syndrome and how it relates to cancer; and* (3) *lack of concern about metabolic syndrome*. Almost all patients reported being unfamiliar with metabolic syndrome prior to being diagnosed with this condition. While patients were familiar with the different risk factors that together constitute metabolic syndrome (i.e., hypertension, dyslipidemia, insulin resistance, and obesity), they were uninformed about this syndrome. Even those who were medically knowledgeable were unaware of metabolic syndrome. For example, one patient said, “*I think a lot of it is education. I’ll be real honest with you. I’ve not really heard a lot about metabolic syndrome. These four criteria that you have here on the screen, obviously I’ve heard of since nursing school—but I didn’t really put them all together for this syndrome. So when you’re talking to people about it, I don’t know that a lot of people are gonna really know what you mean when you say, ‘metabolic syndrome*’” (Sam, 44 years old, central nervous system cancer). Most patients requested additional education on metabolic syndrome and its impact on cancer outcomes, recognizing the importance of this knowledge to effectively caring for themselves through lifestyle modifications. A few patients were unconcerned about their metabolic syndrome and either believed it was being controlled through medication or were concerned more about their cancer diagnosis than about their metabolic conditions.

### Theme 2: Patients’ Attitudes about and Approaches to Managing Metabolic Syndrome

Patients’ attitudes about managing metabolic syndrome were centered on making lifestyle changes and included (1) *lifestyle change is priority*; (2) *motivation to change lifestyle: diagnosis as wake-up call and self-actualization by actively leading their lifestyle change*; and (3) *medications as adjuncts to lifestyle change*. Patients were clear that the means to address their metabolic conditions was to adopt lifestyle modifications to improve their long-term health. This attitude was based on patients’ views and experiences that without lifestyle modifications, any other approaches to address underlying metabolic conditions, for example, weight gain through bariatric surgery or medication use only, would only result in short-term gains. Lifestyle modifications were necessary to sustain long-term changes. For example, one participant said: “*They [providers] didn’t care how it was focusing on the scale number, versus really telling you how you could be the healthiest… The outcome or what the goal should be is feeling better. Feeling healthier. Being healthier. It’s not a matter of losing weight or a number on the scale. It’s a matter of feeling well and maintaining that healthy lifestyle…on the scale, I still weigh 142, and I was thinking, ‘That’s horrible after all this.’ Then it was one of the nutritionists that said, ‘Actually, that is not bad. You’re eating healthy and everything.’ See, if I’m just going with those numbers, that would’ve been one thing, but she was there to say, ‘Oh, no, you’re within that certain amount*. *The goal is you need to be healthy*’” (Ryan, 67 years old, gynecologic cancer).

Many patients stated that receiving a diagnosis of diabetes or cancer served as what they termed a wake-up call, to motivate them to change their lifestyle. These patients understood the importance of behavioral changes to address risk factors contributing to metabolic syndrome and cancer, ultimately improving prognosis. Others expressed that their experience of being diagnosed with cancer motivated them to embark upon substantial lifestyle change and self-actualization to reach their potential. In fact, many patients preferred not to take medications to treat metabolic syndrome, except as an adjunct to behavioral changes. Several even stated that taking medications for metabolic syndrome motivated them to make lifestyle changes to reduce their reliance on medications, as in this example: “*The fact that I know that I don’t like to take medicine, and them adding another medication was like, okay, that’s it. That made it really easy. I don’t want you guys to keep adding different medications for different reasons*. *The best thing to do is to lose weight*” (Dylan, 56 years old, head and neck cancer). Medications were seen as being necessary in the short term until symptoms and conditions improved, but patients described their desire to eventually eliminate medications by incorporating behavioral changes for long-term health.

### Theme 3: Patients’ Capacity and Limitations with Respect to Managing Metabolic Syndrome

Understanding the factors that patients identified as either aiding or limiting the management of their metabolic conditions is an essential step in developing a patient-centered intervention plan. Patients identified both (1) *supports to making lifestyle changes, including* a) *identifying eating strategies to support healthy eating* and b) *family support for diet and exercise changes*, and (2) *challenges to making lifestyle changes*, including a) *financial limitations*, b) *cancer-related weight loss*, and c) *cancer treatment side effects*. Patients were generally well informed about what constitutes a healthy diet and physical exercise program and what worked and did not work personally for them. Many had identified and established routines to encourage healthy behaviors, such as not eating past satiation, not keeping unhealthy snacks at home, meal planning, daily exercise schedules, and low-impact workouts. Family members could serve as either a primary barrier to or a facilitator of patients’ lifestyle changes, depending upon whether family members also adopted the diet or exercise program. One patient noted the value of having family members as motivating exercise buddies: “*It does help to have an exercise buddy.…My daughter joined the Y. Then my granddaughter, who’s in her early 20s, has also joined the Y.…I have somebody with me most of the time, I guess, that I’m at the Y. Which does help. It does help to have somebody else. Plus, when my daughter’s there, she’s like, “Faster, faster, faster*” (Alex, 72 years old, gynecologic cancer).

### Theme 4: Patient-Led Care

Patients described what they needed from their medical team to better manage their metabolic conditions as well as their preferences regarding treatment, which included: (1) *communication: clear, direct communication from medical providers and practical communication tools*; (2) *collaborative care: understanding and respecting patients’ autonomy, attitudes, needs, and preferences*; and (3) *coordination of care among members of the patient’s medical team*. Patients valued clear and direct communication with their team concerning their metabolic conditions. In addition, patients wanted a collaborative, team approach to their care, centered on patients’ needs, preferences, and autonomy. For example, one patient said, “*It’s been the team together that has helped me feel like I could ask the questions that I needed and get the education and support that I needed to do what I needed to do. That’s very good. Allowing the patient to have the resources that they need to take control. In other words, ultimately, the doctor can be there and prescribe medicine and everything, but the patient has to take control of making the decision on this. I’m gonna do this*” (Ryan, 67 years old, gynecologic cancer).

### Theme 5: Tailored Intervention Plans

Most patients adopted an active approach to managing their metabolic conditions, seeking partnerships with their medical team focused on: (1) *co-creation of tailored intervention plans*; (2) *offering instructions tailored to patients’ needs, resources, and support*; and (3) *regular monitoring, feedback, and plan adjustment*. Patients were aware that many individual differences could influence intervention plans, necessitating a flexible approach rather than a standardized treatment approach for each patient. Patients commonly reported needing practical and structured instructions on lifestyle modifications, including nutritional recipes, specific physical exercises, and what to order when eating out. Practical information and guides that could assist patients in their everyday management of metabolic syndrome were highly valued. Particularly important to patients was the development of a structured plan that included regular monitoring and evaluation. Patients noted that monitoring could be done online using secure patient portals, to facilitate adjustment of plans as needed. For example, one patient said, “*I think that because there’s different components to metabolic syndrome, I wanna know, ‘Where am I in that scale?’ ‘This is what your main—what the main concern is for metabolic syndrome for you. We’re going to target two things out of the different components of it’.…So, having my team check in on me on a routine basis, I think, is important. Months will go by that I’m like, ‘How am I really doing? I need to check in with somebody.’ Having someone check in with me, and see where I’m at, and see what they can do to help me. I think it would be great, definitely*” (Parker, 58 years old, head and neck cancer).

## DISCUSSION

In this qualitative study of patients with cancer and metabolic syndrome, we found that patients had limited knowledge of metabolic syndrome and its consequences related to cancer. Most desired additional information and education, although a few expressed a focus on their cancer trajectory over metabolic health. In addition, many patients reported that their cancer or diabetes diagnosis served as a wake-up call, engendering resilience and motivation to make lifestyle modifications a priority. Many patients were willing to take medications as an adjunct to achieving their larger goals. Importantly, patients expressed a strong interest in developing a personalized and holistic care plan focused on a healthy lifestyle rather than simply achieving weight loss. Patients wanted a care plan based on their preferences and capabilities. Patients wanted clear bidirectional communication with their providers, opportunities for their providers to monitor progress, and coordination of care among members of their medical team.

Our findings corroborate the scant previous literature^31,32^ on cancer patients’ understanding of metabolic syndrome and its relationship to cancer outcomes. Seo et al^31^ found that while 56.8% of participants had heard of metabolic syndrome, their knowledge of the syndrome was poor, and 52.3% wanted further information from their medical provider about this condition. Similarly, Jang et al^32^ found that while 70% of participants had heard of metabolic syndrome, their knowledge of the syndrome was poor, and 64.3% wanted further information from their medical provider about this condition. However, whereas Seo et al.^31^ hypothesized that participants’ focus on their cancer progression rather than metabolic syndrome drove their lack of knowledge about this condition, our findings show the opposite, that only a few participants prioritized their cancer progression over metabolic syndrome.

It is crucial to understand patients’ informational needs regarding metabolic syndrome given the high prevalence of metabolic syndrome among cancer patients, which ranges from 24–60% depending on cancer type,^8–10^ and the increased risk of cancer-specific mortality associated with metabolic syndrome.^8,11–14^ Furthermore, common cancer treatments such as surgery, radiotherapy, chemotherapy, and hormonal therapy can induce metabolic syndrome,^33^ worsening patients’ long-term outcomes.

Informing patients about metabolic syndrome is essential in equipping them with the tools they need to manage this condition.^34^ Thus, the development of education resources for patients is essential. In addition, given that existing national oncology recommendations have primarily focused on obesity and weight loss^17–20^ and neglected the larger context of overall health promotion for patients, new evidence-based education and practice guidelines for providers that are informed by and focused on patient-centered health outcomes, such as the guidelines by Wharton et al.,^35^ will be key to addressing metabolic syndrome. A 2018 American Society of Clinical Oncology (ASCO) survey of the oncology workforce showed that providers may find it difficult to incorporate lifestyle Modifications into patient treatment plans because of lack of education on these topics, lack of time, or lack of appropriate programs to which to refer patients.^20^ Development of new education and practice guidelines will help providers overcome these barriers. The ASCO survey also showed that providers perceive resistance from patients to making lifestyle changes^20^, which could make providers reluctant to initiate these discussions. However, findings from our current study suggest that patients are open and ready to receive information and collaborate with providers to identify personalized lifestyle Modifications.

Most patients identified lifestyle Modifications as their preferred approach to managing metabolic syndrome, with medication use being supplementary if and when required. Many stated that receiving a cancer or diabetes diagnosis served as a wake-up call, spurring them to take charge of their health and implement a self-actualization plan, despite various challenges during their cancer trajectory. Patients adopted a committed attitude to persevering through challenges in order to develop flexible action plans that fit their capabilities. Recent literature aligns with our study findings, showing that cancer survivors’ needs and challenges regarding diet and physical exercise vary over the course of their survivorship,^36^ signaling the importance of provider support throughout the cancer trajectory. In a population-based prospective cohort study of 1696 breast cancer survivors, exercise participation and duration increased from 6 months after diagnosis to 18 and 36 months after diagnosis and favored lower-impact activities such as walking.^9^ Findings from other studies showed that the prevalence of metabolic syndrome at 5 years after cancer diagnosis was lower among survivors who participated in exercise for least 30 minutes every day (OR 0.69, 95% CI 0.48–0.98) than among survivors who reported no exercise, supporting previous research on the benefits of physical exercise for those with metabolic syndrome.^37–39^ A recent study demonstrated that a lifestyle intervention based on provider coaching benefited patients by increasing self-efficacy, goal setting, and self-monitoring of results.^40^ Provider coaching was also associated with perceived increased family and provider social support to sustain behavioral changes.^41^ These studies highlight the importance of collaborative relationships between providers and patients, which ultimately empower patients to make healthy lifestyle changes.^42^

We found that patients wanted to engage with their medical team and participate in a personalized treatment plan for their metabolic health issues and lifestyle modifications. This would be in line with a previous call to action to use a patient-centered model within a disease-illness framework to manage obesity.^43^ Patients requested clear communication and monitoring plans with their medical providers. Previous studies have shown that cancer survivors who perceive a lack of support from healthcare providers may experience uncertainty in how to implement lifestyle modifications.^44^ This indicates that patients with cancer would benefit from providers who employ open and direct communication styles. Patients also expressed a desire to collaborate with providers about treatment decisions. In one cluster-randomized clinical trial of shared decision-making in patients with diabetes, patients in the shared decision-making group had higher rates of achieving treatment targets for hemoglobin A1c, blood pressure, and total cholesterol after 24 months compared to patients in the usual care group.^45^ Providers need support and guidance from oncology organizations to implement shared discussions and develop true collaboration to reach agreement about complicated health decisions.^46^

Given the likelihood of inherent biases of self-selection, our study results may not be applicable to cancer patients with metabolic syndrome who did not participate. Additionally, our study reflected the experiences of mostly female, non-Hispanic White, English-speaking cancer survivors with solid tumors. Future research should examine racially and ethnically diverse cancer patients’ experiences of managing metabolic syndrome. Our findings may not be applicable to patients who differ from our study population, such as those currently receiving anticancer therapy.

We believe that our study’s strength lies in its focus on patient experiences as described by patients themselves.^22,47,48^ Learning about patients’ perspective allows providers to understand patients’ knowledge and gain from their wisdom and advice.^49^ Patient’s consistent responses and emphasis on patient-centered and personalized approaches to managing metabolic syndrome provide us with valuable information for updating clinical guidelines. Methodologically, use of a hybrid, inductive/deductive thematic analysis is another strength of this study. Prior research has demonstrated the validity of hybrid, inductive/deductive thematic analysis.^50^ Combining these two approaches serves to overcome the particular weakness of each, while supporting their respective analytic strengths. Inductive methods are faulted for being more susceptible to researcher bias but also are prized as data-driven and capable of generating new ideas and theories. Deductive methods that use predetermined codes can fail to capture important information shared by participants but also are usually developed through consultation of extant literature and knowledge and thus less prone to bias. In this study, various methods were used to safeguard against the potential for researcher bias. For example, each transcript was independently double- or triple-coded and was then reviewed and refined through team coding. Lastly, the research team discussed and developed findings throughout the analytic process to confirm that data supported the final interpretation. Our approach of starting with deductive coding and then moving on to inductive coding allowed for the generation of themes that were ultimately driven by the data and grounded in patients’ lived experiences. The integration of inductive and deductive methods yielded a more balanced and comprehensive understanding of the data.^51^

Our study findings have relevance for oncology providers treating patients with cancer and metabolic syndrome at any point in the cancer journey. Improving the metabolic health of patients with cancer has a direct impact on their cancer-specific survival.^8,11–14,52^ Our study findings may also be useful to primary care providers who care for patients with metabolic syndrome after their active cancer treatment; such providers may be well positioned to offer guidance about lifestyle Modifications.

In summary, our study findings showed that patients with metabolic syndrome and cancer need a collaborative, patient-centered, and personalized care plan. Future work is needed to design educational interventions about metabolic syndrome for patients with cancer. This study also contributed towards, and highlighted the necessity of, updating clinical guidelines for providers that are informed by patients’ perspectives and preferences. Collaborating with patients to identify which lifestyle modifications they would be interested in pursuing is essential to creating a personalized approach. Implementation of practical management and monitoring strategies for patients with cancer and metabolic syndrome along with longitudinal assessment of their metabolic endpoints will be critical to assess the impact of interventions.

## Figures and Tables

**Table 1.** Participant Characteristics (N = 19)^a^

Characteristic	Value
Age, mean (SD), y	64.6 (13.4)
Gender	
Female	12 (63)
Male	7 (37)
Race	
Asian	2 (11)
Black	3 (16)
White	12 (63)
Two or more races	2 (11)
Ethnicity	
Hispanic	3 (16)
Non-Hispanic	16 (84)
Born in the US	
Yes	16 (84)
Marital status	
Married	15 (79)
Separated	2 (11)
Divorced	1 (5)
Widowed	1 (5)
Education	
High school diploma	8 (42)
Bachelor's degree	6 (32)
Master's degree	2 (11)
Doctoral degree	2 (11)
Professional degree	1 (5)
Primary cancer diagnosis	
Gynecologic	5 (26)
Genitourinary	4 (21)
Colorectal	3 (16)
Breast	2 (11)
Head & neck	2 (11)
Central nervous system	1 (5)
Lung	1 (5)
Non-Hodgkin lymphoma	1 (5)
Systemic cancer treatment	
Actively receiving	5 (26)
Completed	14 (74)

a Values in table are number of patients (percentage) unless otherwise indicated.

**Table 2.** Themes, Associated Categories, and Selected Exemplary Quotes

Themes				Categories			Selected Exemplary Quotes
Knowledge of metabolic syndrome		Patients' understanding of metabolic syndrome		Unfamiliarity with metabolic syndrome			"[Metabolic syndrome] was never fully explained to me.this is what this is, and this is what you can do to control it." (Parker, 58 years old, head & neck cancer)
				Desire for education about metabolic syndrome and how it relates to cancer			"There's not a lot of education on exactly what is the best for you. I think that was a big game changer for me. I had a desire to, first of all, find out — Okay, especially not only with the metabolic syndrome but with my cancer and everything. I found out that a lotta this is connected. What was the best thing for me to do nutrition-wise? That really helped me out. I think education would be the number one thing." (Ryan, 67 years old, gynecologic cancer)
				Lack of concern about metabolic syndrome			"Let me put this in context. I'm really not worried about metabolic syndrome; my chief concern is treating the cancer. Something's going to kill me. I'm 84 years old, and this is an end game." (Taylor, 84 years old, genitourinary cancer)
Attitudes about lifestyle modifications		Patients' attitudes about and approaches to managing metabolic syndrome		Lifestyle change is priority			"I think the most important thing is the lifestyle adjustment." (Jodie, 66 years old, gynecologic cancer)
				Motivation to change lifestyle		Diagnosis as wake-up call	"For me, cancer was a really big wake-up call. When you're faced with life and death you see things differently.it scares the bejabbers out of you. In my mind I didnť have a choice. It was either do that [change lifestyle] or I risk the chance of dying and I've got four kids at home. I'm not willing to risk my chance of dying" (Drew, 52 years old, gynecologic cancer)
						Selfactualization by actively leading their lifestyle change	"It was a personal commitment that I wanted to make a difference in my own lifestyle— what is it that I wanna do in my life? Once, if that is clearly defined, then you put in action plans which are needed to meet that goal....I donť think any limitations have any impact on it....Thaťs what you have to do. Once you make the commitment you stick to it." (Jessie, 83 years old, genitourinary cancer)
				Medications as adjuncts to lifestyle change			"Anything that I can do to help eliminate [medications] is a huge plus in my book....I think the most important thing is you try to change your eating habits and exercise....I never thought that those were gonna be truly important in your life, but it is. Iťs made a huge difference in my life.if I end up doing all that and trying to fix it on my own and I still canť, I have to understand that iťs hereditary and iťs [medication] part of what I have to do for the rest of my life....Iťs important to have some kind of control that you can control the situation a bit by eating better and exercising, I think it goes a long way. I'm a true believer in that." (Parker, 58 years old, head & neck cancer)
	Patients' capacity and limitations with respect to managing metabolic syndrome			Supports to making lifestyle changes	Identifying eating strategies to support heathy eating		"Now, we live in an area with several people all around us that are very social, and there's always events going on. I guess thaťs one of the biggest barriers, is that you're not—unless you physically bring the right food all the time, iťs not always available. You either have to eat before you go or after you come back and that type of thing." (Jodie, 66 years old, gynecologic cancer)
					Family support for diet and exercise changes		"Exercise, adding the exercise, tryin' to get mobility, and the military has given me some exercises that I can do thaťs low impact on my knees and my hips....My mom, she went from a size 28 to a 6, 8 now....A big part of what she does daily is exercise....She's very encouraging. " (Dylan, 56 years old, head & neck cancer)
				Challenges to making lifestyle changes	Financial limitations		"Right now, I would say iťs more economics, like everything's getting more expensive. Right now, iťs like, you cannot really pick what you want, like organic this or organic that, so that would be a challenge." (Kai, 57 years old, gynecologic cancer)
					Cancer-related weight loss		"My problem, now, is not that—I'm never hungry. Most of the times, I make myself eat because I take medication, and bein' a cancer patient, I need to keep weight on. I try to eat even when I really donť want to. The food tastes—still tastes good. Iťs just my appetite. I really donť want it. I donť have a weight problem. I have a problem with keepin' the weight I have on." (Riley, 68 years old, lung cancer)
					Cancer treatment side effects		"Mobility is a little hard because the enfortumab really did produce a significant peripheral neuropathy both afferent and efferent. I did a lot of falling early on." (Taylor, 84 years old, genitourinary cancer)
Preferences for lifestyle interventions	Patient-led care			Communication	Clear, direct communication from medical providers		"I'm all about people just being straightforward and honest and saying, 'Here's the issue, and here's what we need to do to help fix it,' and so just kinda straightforward, shoot it to me straight, and tell me what I can do to make it better and—probably works best for me." (Kai, 57 years old, gynecologic cancer)
					Practical communication tools		"I really like the MyChart because that gives me the ability to communicate with the nurses and provide that feedback and get feedback from them." (Quinn, 74 years old, non-Hodgkin lymphoma)
			Collaborative care		Understanding and respecting patients' autonomy, attitudes, needs, and preferences		"When I was first in the hospital and I was diagnosed with diabetes because they diagnosed me when they found out I had cancer, they went straight to insulin versus trying to give me time to get on pill form, and I had an issue with that. 'Cause I just didnť wanna jump right into insulin. But when they let me go home and I told them I didnť wanna be on insulin, I wanted to be on something pill form, and they did it. (Drew, 52 years old, gynecologic cancer)
			Coordination of care		Among members of the patienťs medical team		"The team was working together, I guess would be a good way to say. Team partnership on your whole health. I was seen by the—started out with my cancer doctors. Then they referred me to the cardiologist, the endocrinologist, and then to Dr. X, who also then got me into integrated medicine." (Ryan, 67 years old, gynecologic cancer)
Co-creation of tailored intervention plans			Offering instructions tailored to patients' needs, resources, and support				"An achievable goal for where I am in my health because not everybody can do the same. Not everybody's in the same process of healing. Individualized. Thaťs what we're coming to. Again, an individualized plan or goal for you where you are may not be the same as for Mr. X over here, who has the same, maybe, diagnosis but is in a different place." (Alex, 72 years old, gynecologic cancer)
			Regular monitoring, feedback, and plan adjustment				"Iťs like I take the blood pressure measurement—the blood level—sugar level— every morning. Then I send that periodically to Dr. Y so that she can see the trend....I try to do it once a month or whatever. Iťs my way of giving her feedback that what she's asked me to do, or what I needed to do, is working. It doesnť require her to reply back. Iťs just so that she can see that we're going in the right direction....That shows me that iťs working. If I didnť see something that showed numbers that show that what you're doing is making a difference, then I probably wouldnť be doing it. Iťs the feedback to me that keeps me going." (Quinn, 74 years old, non-Hodgkin lymphoma)

## Data Availability

The datasets generated and analyzed during the current study are not publicly available because the data for this study are from a qualitative dataset but may be available from the corresponding author on reasonable request.
